# Argon protects against hypoxic-ischemic brain injury in neonatal rats through activation of nuclear factor (erythroid-derived 2)-like 2

**DOI:** 10.18632/oncotarget.8241

**Published:** 2016-03-21

**Authors:** Hailin Zhao, Sian Mitchell, Sarah Ciechanowicz, Sinead Savage, Tianlong Wang, Xunming Ji, Daqing Ma

**Affiliations:** ^1^ Department of Surgery and Cancer, Section of Anaesthetics, Pain Medicine and Intensive Care, Faculty of Medicine, Imperial College London, Chelsea & Westminster Hospital, London, UK; ^2^ Department of Anesthesiology, Xuanwu Hospital, Capital Medical University, Beijing, China; ^3^ Department of Neurosugery, Xuanwu Hospital, Capital Medical University, Beijing, China

**Keywords:** argon, perinatal hypoxic-ischaemic encephalopathy, Nrf2

## Abstract

Perinatal hypoxic ischaemic encephalopathy (HIE) has a high mortality rate with neuropsychological impairment. This study investigated the neuroprotective effects of argon against neonatal hypoxic-ischaemic brain injury.

*In vitro* cortical neuronal cell cultures derived from rat foetuses were subjected to an oxygen and glucose deprivation (OGD) challenge for 90 minutes and then exposed to 70% argon or nitrogen with 5% carbon dioxide and balanced with oxygen for 2 hours.

*In vivo*, seven-day-old rats were subjected to unilateral common carotid artery ligation followed by hypoxic (8% oxygen balanced with nitrogen) insult for 90 minutes. They were exposed to 70% argon or nitrogen balanced with oxygen for 2 hours. *In vitro*, argon treatment of cortical neuronal cultures resulted in a significant increase of p-mTOR and Nuclear factor (erythroid-derived 2)-like 2(Nrf2) and protection against OGD challenge. Inhibition of m-TOR through Rapamycin or Nrf2 through siRNA abolished argon-mediated cyto-protection. *In vivo*, argon exposure significantly enhanced Nrf2 and its down-stream effector NAD(P)H Dehydrogenase, Quinone 1(NQO1) and superoxide dismutase 1(SOD1). Oxidative stress, neuroinflammation and neuronal cell death were significantly decreased and brain infarction was markedly reduced. Blocking PI-3K through wortmannin or ERK1/2 through U0126 attenuated argon-mediated neuroprotection.

These data provide a new molecular mechanism for the potential application of argon as a neuroprotectant in HIE.

## INTRODUCTION

Perinatal hypoxic-ischaemic encephalopathy (HIE) is one of the largest contributors to neonatal brain injury with subsequent poor developmental outcome [1]. It has been shown that between 20-50% of asphyxiated infants die in the neonatal period, with 25% of survivors suffering neuropsychological deficits such as cerebral palsy, learning disabilities or epilepsy [2]. The encephalopathy caused by the ischaemia is defined initially by the primary injury, and is exacerbated by further processes involving apoptosis leading to brain damage [3]. The outcome of a hypoxic ischaemic episode is influenced by several factors such as the duration and severity of insult to the brain, gestational age, presence of seizures and associated infectious, metabolic and traumatic problems [2]. Given the incidence rate and huge impact on families, including the need for long-term multi-disciplinary care [4], prevention strategies are continually searched for [5].

Previous treatment for HIE included drugs that reduced cerebral odema such as osmotic diuretics [5]. Current avenues for neuroprotection involve hypothermia [6], including total body cooling [7], with erythropoietin therapy also investigated [8]. However, treatments are far from optimal, therefore there is much scope for the discovery of novel strategies for HIE.

Reactive oxygen species (ROS) have been implicated to play a key role in the pathogenesis of HIE and induces cell death via oxidation of membrane lipids and proteins [9]. Nuclear factor (erythroid-derived 2) factor 2 (Nrf2) mediates cellular protection against oxidative stress [10] and has become the focus of new neuro-protective strategy. Under oxidative stress, Nrf2 proteins translocate into the nucleus. Nrf2 binds to antioxidant response elements (AREs) of DNA, and stimulates transcription of antioxidant proteins. These include gluthatione S-transferases (GSTs) and NAD(P)H: quinone oxidoreductase 1 (NQO1)[11]. Phosphatidylinositol 3-kinase (PI3K)/Akt pathway (PI3K/Akt pathway) has been shown to regulate the Nrf-2 pathway [12].

Xenon, a noble gas, is a neuroprotectant with no known toxicity, has rapid onset and few side effects unlike other inhaled gases [13]. Its use is currently limited due to expense [3]. Recently, another noble gas, argon, has also been shown to have neuroprotective effects [8, 14]. In an *in vitro* model, it has been demonstrated that argon is neuroprotective in organotypic hippocampal cultures after oxygen glucose deprivation [15]. In addition, argon was protective against hypoxic injury and injury caused by cisplatin and gentamicin in rat cochlear cultures [16]. In an *in vivo* study, normobaric argon increased the survival rates of rats exposed to changing degrees of hypoxia [17]. The aim of this study was to investigate whether argon affects neuronal cell death and inflammation after hypoxic-ischaemic insult both *in vitro* to oxygen glucose deprivation induced injury and *in a* rat model of neonatal asphyxia. In addition, the underlying molecular mechanisms including the role of Nrf2 were also explored.

## RESULTS

### Argon exposure induced up-regulation of PI-3K, Erk1/2 and p-mTOR in the cultured rat cortical neurons

**Figure 1:**
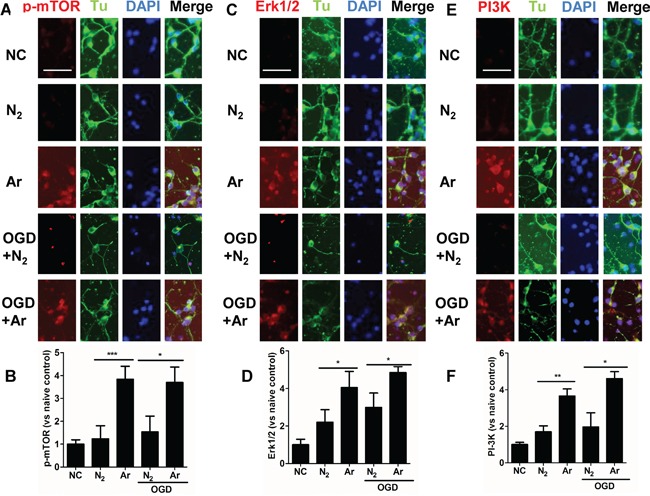
Enhanced expression of PI-3K, ERK1/2 and p-mTOR in cultured cortical neuronal cells after argon exposure Rat neuronal cell culture was exposed to argon gas (70% Ar and 5% CO_2_ balanced with O_2_) or nitrogen gas (70% N_2_ and 5% CO_2_ balanced with O_2_) for 2 hours and then room air for 24 hours. For the OGD experiment, rat cortical neuronal cell culture were given oxygen glucose deprivation (OGD) for 90 minutes and then exposed to argon gas (70% Ar and 5% CO_2_ balanced with O_2_) or nitrogen gas (70% N_2_ and 5% CO_2_ balanced with O_2_), for 2 hours and then room air for 24 hours. **A.** Dual immunolabelling of α-Tubulin (Tu, green fluorescence) and p-mTOR (red fluorescence) **B.** Fluorescent intensity of p-mTOR at 4 hours after gas exposure. **C.** Dual immunolabelling of α-Tubulin (Tu, green fluorescence) and Erk1/2 (red fluorescence) **D.** Fluorescent intensity of Erk1/2 at 4 hours after gas exposure. **E.** Dual immunolabelling of α-Tubulin (Tu, green fluorescence) and PI-3K (red fluorescence) **F.** Fluorescent intensity of PI3K at 4 hours after gas exposure. Cell nuclei were counter-stained with DAPI (blue). Data are Mean ± SD. n = 8. *p<0.05 and **p<0.01 and ***p<0.001, scale bar: 50μm. NC: naïve control.

To assess the role of PI3K/Akt pathway and MAPK pathway in cultured neurons following exposure to argon, the change in the expression of PI3K, ERK1/2 and p-mTOR were firstly assessed through immunofluorescent staining. The immunofluorescent intensity of PI3K, Erk1/2 and p-mTOR was markedly enhanced when the cells were exposed to argon (Figure 1A-1F). Enhanced expression was also observed in argon treated neuronal cell challenged with OGD (Figure 1A-1F).

### Argon reduced neuronal injury induced by OGD *in vitro* through enhanced Nrf2 expression

**Figure 2:**
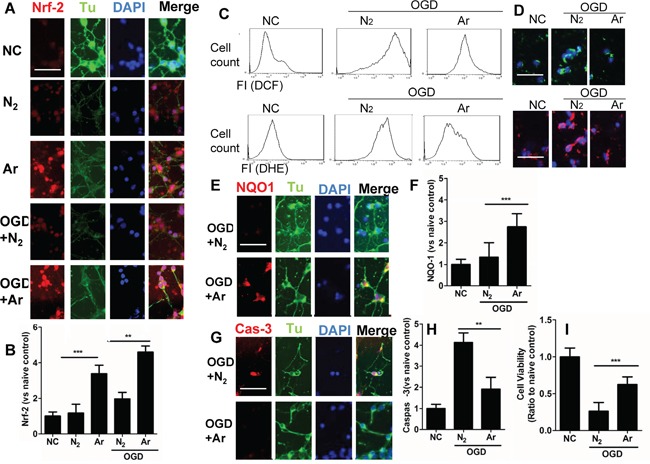
Effect of argon exposure on cortical neuronal cell death after oxygen glucose deprivation (OGD) Rat neuronal cell culture were exposed to argon gas (70% Ar and 5% CO_2_ balanced with O_2_) or nitrogen gas (70% N_2_ and 5% CO_2_ balanced with O_2_) for 2 hours and then room air for 24 hours. For the OGD experiment, rat cortical neuronal cell cultures were given OGD for 90 minutes and then exposed to argon gas (70% Ar and 5% CO_2_ balanced with O_2_) or nitrogen gas (70% N_2_ and 5% CO_2_ balanced with O_2_), 2 hours and then room air for 24 hours. **A.** Dual immunolabelling of α-Tubulin (Tu, green fluorescence) and Nrf2 (red fluorescence) **B.** Fluorescent intensity of Nrf2 at 4 hours after gas exposure. Cellular ROS (O.- stained with DHE) and (H_2_O_2_ stained with DCF) were assessed by **C.** Flow cytometry and **D.** Fluorescent microscopy. **E.** Dual immunelabelling of α-Tubulin (green fluorescence) and NQO-1 (red fluorescence) **F.** Fluorescence intensity of NQO-1 at 4 hours after gas exposure. **G.** Dual immunelabelling of α-Tubulin (green fluorescence) and caspase-3 (red fluorescence) **H.** Fluorescent intensity of caspase-3 at 4 hours after gas exposure. **I.** Cell viability assessed by MTT assay. Data is expressed as Mean ±SD. (n=8, **p<0.01 and ***p<0.001). Scale bar: 50μm. NC: naïve control, IC: injury control. Hy:Hypothermia.

OGD-induced injury provokes neuronal death through oxidative stress [18]. The nuclear factor erythroid 2-related factor 2 (Nrf2) is a critical regulator of cellular resistance to oxidants [11]. Studies have shown that activation of PI-3K pathway or ERK1/2 pathway leads to enhanced production of Nrf2 through p-mTOR [19]. Increased expression and translocation of Nrf2 into nuclei were also observed after argon exposure (Figure 2A and 2B), indicating activation of the antioxidant system. Indeed, expression of its down-stream effector NAD(P)H dehydrogenase (quinone 1) (NQO-1), which acts as a superoxide scavenger [20], was augmented during OGD challenge (Figure 2E and 2F). 4 hours after treatment, production of ROS was assessed by flow cytometry (Figure 2C and 2D). Argon treatment significantly reduced production of ROS after OGD challenge (Figure 2C and 2D), and consequently, the expression of cleaved caspase-3 was decreased (*p*<0.05, argon vs N_2_), which indicated enhanced cell survival through OGD challenge after argon treatment (Figure 2G and 2H). The external morphology of neurons remained relatively unaltered 4 hours after OGD (Figure 2G and 2H). The neuronal cell viability was assessed by MTT assay at 24 hours after OGD exposure. Argon significantly increased the cell viability in OGD-exposed neurons (P<0.05, argon vs N_2_) (Figure 2I). These results in combination give strong evidence that argon treatment conferred protection against OGD-challenged neurons.

### Inhibition of m-TOR and Nrf2 abolished argon-mediated cyto-protection in cultured rat cortical neurons

**Figure 3:**
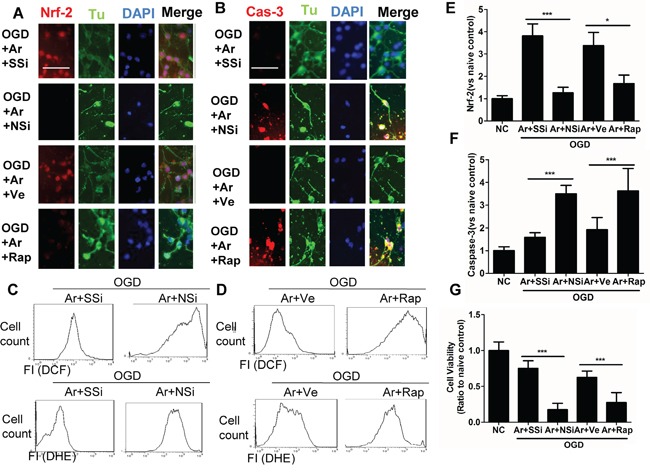
Inhibition of Nrf2 or p-mTOR attenuated the cytoprotective effects conferred by argon in cortical neuronal cells after OGD deprivation Rat cortical neuronal cells transfected with negative control scramble siRNA or Nrf2 siRNA for 6 hours, prior to OGD treatment, or were treated with p-mTOR inhibitor rapamycin, after OGD treatment. Rat cortical neuronal cell culture were given OGD for 90 minutes and then exposed to argon gas (70% Ar and 5% CO_2_ balanced with O_2_) or nitrogen gas (70% N_2_ and 5% CO_2_ balanced with O_2_) for 24 hours. **A.** Dual immunolabelling of α-Tubulin (green fluorescence) and Nrf2 (red fluorescence), **B.** Dual immunelabelling of α-Tubulin (green fluorescence) and caspase-3 (red fluorescence). **C.** Cellular ROS (O.-) and **D.** (H_2_O_2_) are assessed by flow cytometry, **E.** Fluorescent intensity of Nrf2 at 4 hours after gas exposure. **F.** Fluorescent intensity of cleaved caspase-3 at 4 hour after gas exposure. **G.** Cell viability assessed by MTT assay. Data is expressed as Mean ±SD. (n=8, *p<0.05 and ***p<0.001). Scale bar: 50μm. NC: naïve control, OGD: oxygen glucose deprivation. Ve: Vehicle, Rap: rapamycin, SSi: scramble siRNA, NSi: Nrf2 siRNA.

Upon investigating whether argon-associated neuroprotection was mediated through p-mTOR and Nrf2, we demonstrated a reinstatement of cleaved caspase-3 expression of OGD-induced neurons when m-TOR inhibitor rapamycin was applied. Likewise, when neuronal cells transfected with Nrf2 siRNA were subjected to OGD, the increase was inhibited significantly (Figure 3A, 3B, 3E and 3F). The cultures without inhibitors showed extensive neuronal processes whereas those treated with inhibitors did not after OGD challenge (Figure 3A). The suppression of ROS production was attenuated by either Nrf2 siRNA or rapamycin (Figure 3C and 3D). Consequently, cell survival after OGD challenge was significantly lower after Nrf2 or p-m-TOR was blocked (Figure 3G). These results support the hypothesis that p-mTOR and Nrf2 regulate argon-associated neuroprotection and mediate the resistance against oxidative stress in rat cortical neurons.

### Argon treatment enhanced expression of anti-oxidant enzymes in rat cortex with hypoxic-ischemia injury

**Figure 4:**
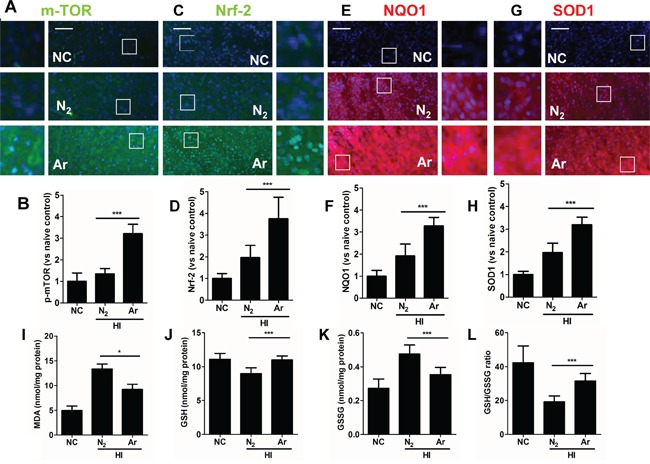
Argon treatment activates anti-oxidative protein expression and reduced oxidative stress in brain cortex with hypoxic-ischaemia injury Rats were given hypoxic ischaemia injury for 90 minutes and then exposed to argon gas (70% Ar balanced with 30% O_2_) or nitrogen gas (70% N_2_ balanced with 30% O_2_) for 2 hours and then room air for 24 hrs. In rat cortex, expression of **A.** p-mTOR (green fluorescence) **B.** Fluorescent intensity of p-mTOR at 4 hours after gas exposure. **C.** Nrf2 (green fluorescence) **D.** Fluorescent intensity of Nrf2 at 4 hours after gas exposure. **E.** NQO-1 (red fluorescence) **F.** Fluorescent intensity of NQO-1 at 4 hours after gas exposure. **G.** SOD-1 (red fluorescence) **H.** Fluorescent intensity of SOD-1 at 4 hours after gas exposure. Cell nuclei were counter-stained with DAPI (blue). **I.** Cortical tissue MDA level. **J.** Cortical tissue GSH level, **K.** Cortical tissue GSSG level. **L.** Cortical tissue GSH to GSSG ratio. Data are means ± SD. n = 8. *p<0.05 and ***p<0.001, scale bar: 50μm. NC: naïve control, HI: hypoxic ischaemia injury.

In parallel with *in vitro* observation, immunofluorescence analysis demonstrated that p-mTOR, Nrf2 and its downstream effectors NAD(P)H dehydrogenase (quinone 1) (NQO1) and superoxide dismutase 1(SOD1) protein expression levels were also increased considerably in hypoxic-ischaemia rat brain cortex after argon treatment, compared with those with nitrogen treatment (Figure 4A-4H). Meanwhile, the content of MDA in ischaemic cortical tissue was detected to examine the oxidative response at 24 hours after HI. MDA levels in hypoxic neurons were also reduced after argon treatment (Figure 4I). When argon was administered after hypoxia, it significantly increased the levels of GSH, reduced levels of GSSG in the developing brain after 24 hours, the overall GSH-GSSG ratio was also increased by this treatment (Figure 4J-4L).

### Argon reduced neuronal cell death and neuro-inflammation in rat cortex after hypoxic-ischaemia

**Figure 5:**
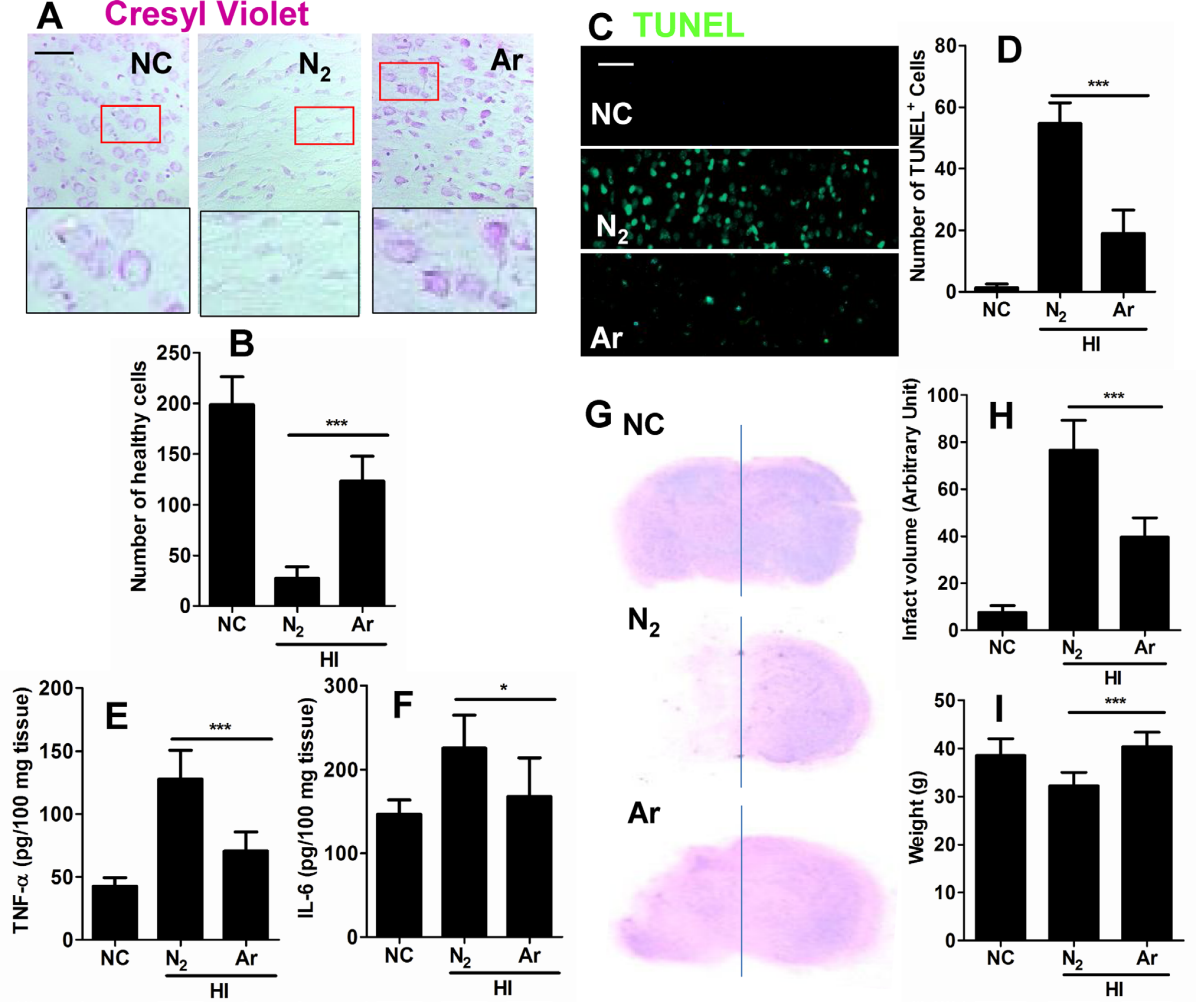
Effect of argon on neuronal cell death and inflammation in brain cortex after hypoxic-ischaemia Rats were given hypoxic ischaemic injury for 90 minutes and then exposed to argon gas (70% Ar balanced with 30% O_2_) or nitrogen gas (70% N_2_ balanced with 30% O_2_) 2 hours and then room air for 24 hrs. **A.** Cresyl violet staining of brain cortex 24 hours after gas exposure. **B.** Number of healthy neuronal cells per x 20 field 24 hours after gas exposure (n = 8). **C.** Tunnel staining at 24 hours after gas exposure. **D.** Number of TUNEL positive neuronal cells per x 20 field 24 hours after gas exposure (n = 8). **E.** Neonatal rat brain cortex tissue TNF-α level 24 hours after gas exposure (n = 8). **F.** Neonatal rat brain cortex tissue IL-6 level 24 hours after gas exposure (n = 8). **G.** Representative brain micrograph 28 days after experiments, stained by cresyl violet. **H.** Infarct volume 28 days after experiments (n = 10). **I.** Body weight of rats 28 days after experiments (n = 10). Data are means ± SD. *p<0.05 and ***p<0.001, scale bar: 50μm. NC: naïve control, HI: hypoxic ischaemia injury.

To investigate whether argon was able to reduce neuronal cell death, cell morphology was assessed 24 hours after HI. Overall increased numbers of healthy cells were observed in the argon treatment group (Figure 5A and 5B). Consistent with this observation, there was a significant reduction in the number of TUNEL+ cells between the nitrogen treated group and the argon treated groups 6 hours after the brain received HI injury (Figure 5C and 5D), demonstrating that argon was able to reduce cell death. Cortical tissue inflammatory cytokines, such as TNF-α and IL-6 were reduced after argon exposure, which indicated that neuro-inflammation was attenuated by the treatment (Figure 5E and 5F). The long-term protective profile of argon treatment was explored. Extensive lesions developed in the cortex in N_2_ plus hypoxia treated groups (Figure 5G). Argon treatment significantly reduced infarction volume, when compared with nitrogen control (Figure 5G and 5H). The pathological change by the argon treatment correlated positively with rat body weight (Figure 5I), with argon exposure associated with increased body weight, when compared with nitrogen control.

### Inhibition of ERK1/2 and PI-3K attenuated the argon-mediated neuroprotection

**Figure 6:**
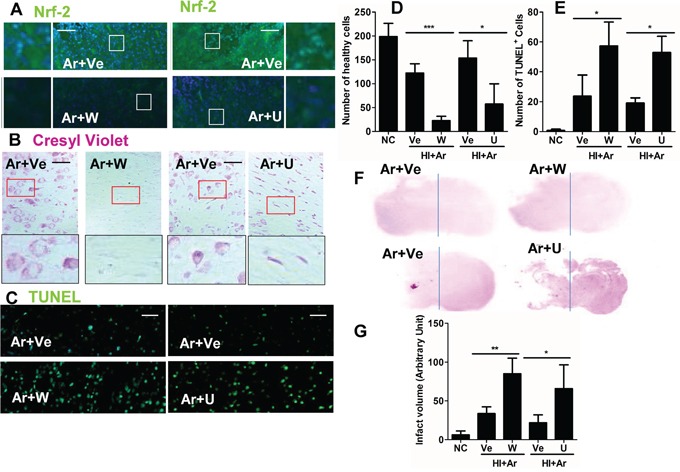
PI-3K and ERK1/2 inhibitors abolished argon-mediated neuroprotection Rats were given hypoxic ischaemic injury for 90 minutes and then exposed to argon gas (70% Ar balanced with 30% O_2_) or nitrogen gas (70% N_2_ balanced with 30% O_2_) for 2 hours and then room air for 24 hrs. PI3K-Akt inhibitor wortmannin and Erk1/2 inhibitor U0126 was administered after hypoxic-ischaemia injury. **A.** Nrf2 expression (green fluorescence) in cortex at 24 hours after gas treatment. **B.** Cresyl violet staining of brain cortex 24 hours after gas exposure. **C.** Tunnel staining at 24 hours after gas exposure. **D.** Number of healthy cells in brain cortex per x 20 field 24 hours after gas exposure. **E.** Number of TUNEL+ neuronal cells in brain cortex per x 20 field 24 hours after gas exposure. **F.** Representative brain micrograph 28 days after experiments, stained by cresyl violet. **G.** Infarct volume 28 days after experiments. Data are means ± SD. n = 8. *p<0.05 and **p<0.01 and ***p<0.001, scale bar: 50μm. NC: naïve control, HI: hypoxic ischemic injury. Ve: vehicle. W: wortmannin, U: U0126.

To determine which pathway argon bought about its neuroprotective effects, wortmannin, a specific PI3K inhibitor and U0126, a specific ERK1/2 inhibitor were utilised. Inhibition of PI-3K and ERK1/2 activities blocked the up-regulation of Nrf2 (Figure 6A) and the neuroprotective effects of argon (Figure 6B-6G). This provides further support that PI-3K and ERK1/2 are positive regulators of the Nrf2 pathway and its neuroprotective effects.

## DISCUSSION

The present studies were undertaken to test the hypothesis that argon exposure would initiate the protective response against HI brain injury in neonatal rats. The transcription factor Nrf2 was found to play a pivotal role in mediating the neuroprotective effects of argon.

**Figure 7:**
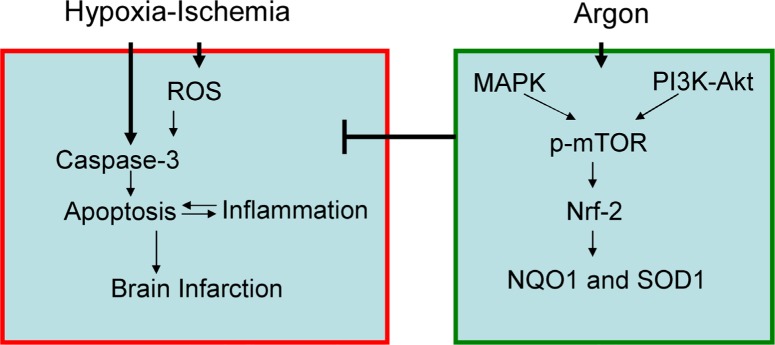
Proposed molecular mechanism for argon-mediated neuroprotection Argon exposure induced activation of PI-3K and ERK1/2 pathway, leading to up-regulation of p-mTOR and Nrf2. Expression of down-stream anti-oxidative effectors, such as NQO-1 and SOD-1 was enhanced, leading to suppression of ROS production in brain cortex after hypoxic-ischaemia. Consequently, neuronal cell death and inflammation were inhibited and brain infarction volume was reduced.

It is only relatively recently that the neuroprotective action of argon has been recognised [21]. Furthermore it has been shown that argon given after excitotoxic or ischaemic insult reduces neuronal cell injury [22]. In this study, argon exposure induced enhanced expression and nuclear translocation of Nrf2 in cultured cortical neuronal cells. In addition, production of reactive oxygen species (ROS) and caspase-3 activation were markedly decreased when argon treatment was given to neurons exposed to OGD. Parallel to *in vitro* observation, our *in vivo* study has also demonstrated that argon exposure to neonatal rats with hypoxic-ischaemic insults decreased the level of MDA product, increased the down-stream effector expression of NAD(P)H dehydrogenase [quinone] 1(NQO1) and superoxide dismutase 1(SOD-1). Consequently, neuronal cell death was suppressed and infarct volume was reduced (summarised in Figure 7).

Our study demonstrates, for the first time, that the neuroprotective mechanisms of argon involve activation of the transcription factor NF-E2-related factor 2 (Nrf2), which is considered to be a mediator of organoprotection by up-regulating the expression of many antioxidant enzymes under oxidative stress [23]. Nrf2 is a basic leucine zipper transcription factor that binds and activates the transcription of antioxidant response element (ARE) [19]. Under normal physiological conditions, Nrf2 is bound to Kelch-like ECH-associated protein 1 (Keap1), leading Nrf2 to ubiquitination and proteosomal degradation [24]. However, under oxidative stress, Keap 1 repression of Nrf2 is inhibited, Nrf2 protein is then translocated into the nucleus and activates its target genes [25]. Activation of Nrf2 has been shown to be mediated through ERK1/2 pathway [26] and PI3k/Akt [27]. The antioxidant genes controlled by Nrf2, include heme oxygenase-1 (HO-1), glutathione S-transferases (GSTs) and NAD(P)H quinone oxidoreductase, which scavenge reactive oxygen species (ROS) and prevent damage by oxidative stress [11]. Recent studies show that Nrf2 mediates protection against neuronal cell death [28] and neuro-inflammation [29]. Consistent with these studies, we observed significant up-regulation of SOD-1 and NQO1 in neonatal rat brain cortex after HI, following treatment with argon.

Previous studies have demonstrated the neuro-protective nature of argon, Ulbrich et al [30] demonstrated argon conferred neuroprotection via an induction of an ERK with critical involvement of HO-1 (heamoxiginase-1) in retinal ganglion cells after ischemia and reperfusion injuries. This is consistent with our study; since HO-1 regulates the anti-oxidative response against cell injury and it is involved in the regulation of the expression and activity of Nrf-2 [31].

Our study also explored the effect of argon exposure on PI3K, Erk1/2 and p-mTOR, which play key roles in many cellular processes such as cell proliferation. P13K activates Akt and then m-TOR [32]. It has been reported that xenon exposure activates the P13K/Akt pathway in neuronal cell cultures [33]. There is also cross talk from PI3K to activate ERK, a ubiquitous cell proliferation and survival enzyme [34, 35]. Previously, it was demonstrated that argon markedly increased expression of ERK, in the microglial cell line, BV-2 and in neuronal and astroglial cell cultures [36]. In this study, PI-3K and ERK expression was increased at 4 hours after gas treatment. Potentially argon works via PI-3K cell signalling cascade as well as ERK, and in addition could also act via crosstalk between P13K and ERK [34]. This is further supported by the use of PI-3K inhibitor wortmannin and ERK1/2 inhibitor U0126 to abolish argon-mediated neuroprotection.

Argon is safe and has no record of toxicity. In our study, argon exposure was effective in promoting neuronal survival after OGD and reducing experimental hypoxic-ischemia injury in neonatal rats. Both argon and xenon have good blood brain barrier penetration [14], which means both have rapid onset. Given that argon is relatively inexpensive compared to xenon, if argon can be shown to be as neuroprotective as xenon, it could be a more economically viable treatment, making it promising for clinical use. Further studies should investigate whether other signalling pathways are also involved in the protective effects of argon. Taken together, this suggests that argon may be useful in treating ischaemic injury in humans.

## MATERIALS AND METHODS

### Primary cortical neuronal cell culture

Cortical neuronal cells were derived from embryonic day 18 Sprague Dawley rat foeti. Cerebral cortices were dissected and trypsinised in 0.25% trypsin for 30 minutes at 37°C. Cells were filtered through a 40μm cell strainer to obtain a single cell suspension, and then resuspended in neurobasal media (Gibco, UK), supplemented with glutamine (25μl/10ml;Sigma Aldrich), B27 (200μl/10ml; Sigma Aldrich) and antibiotic antimycotic solution (penicillin 100U/ml, streptomycin sulphate 100μg/ml, amphotericin B 0.25μg/ml; Fischer Scientific, UK). Cells were cultured in a humidified incubator at 37°C with 5% CO_2_ for 7 days before experiments.

### Gas exposure *in vitro*

For the argon experiments, solutions were prepared by bubbling pure gases (nitrogen or argon; Air Products, Crewe, United Kingdom) through fine sintered-glass bubblers in Drechsel bottles (Pegasus, Guelph, Canada) filled with culture medium. Cells were kept in purpose-built, airtight, temperature-controlled, cell-culture chambers. These were pre-filled with the desired concentration of argon after the culture medium had been replaced by argon-bubbled medium. In the post-OGD period, cells were returned to a normoxic incubator containing nitrogen or argon (75% argon or nitrogen, and 5% carbon dioxide balanced with oxygen) for 24 hours at 37°C.

### Oxygen glucose deprivation (OGD)

The OGD procedure has been described on previous protocols [37]. Briefly, Neurobasal media (Gibco, UK) was replaced with a sterile and deoxygenated balanced salt solution pre-warmed to 37°C, pH 7.4 with ionic composition (NaCl 116mm, KCl 5.4mm, MgSO_4_ 0.8mm, NaH_2_PO_4_ 1.0mm, CaCl_2_ 1.8mm, NaHCO_3_ 26mm, glucose 5mm). Before use, pure nitrogen or argon gas was bubbled through for 20 minutes using Drechsel bottles to remove oxygen from the solution. After replacing the media, the cultures were incubated for 90 minutes in an airtight gas chamber filled with treatment gas excluding oxygen. For the nitrogen group, the cultures were exposed to 95% N_2_ and 5% CO_2_ and for the argon group, cultures were exposed to 70% argon, 25% N_2_ and 5% CO_2_. After 90 minutes, the media was replaced with neurobasal media and was incubated again for a further 24 hours at 70% argon, 25% O_2_ and 5% CO_2_ for the argon group and 70% N_2_, 25% O_2_ and 5% CO_2_ for the nitrogen group.

### Animals

7-day old Sprague-Dawley rat pups were purchased from Harlan UK and housed in the animal facilities in Chelsea and Westminster hospital campus, Imperial College London. All procedures were conducted in accordance with the United Kingdom Animals (Scientific Procedures) Act of 1986. All animal procedure are compliant with the ARRIVE guidelines for reporting animal experiments [38].

### Rat hypoxic-ischaemic brain injury

An *in vivo* rat model of neonatal hypoxic-ischaemia (HI) was used for this study. Right common carotid artery ligation was performed on 7-day-old Sprague-Dawley rats under surgical anaesthesia (5% isoflurane induction, 2% isoflurane maintenance), as previously described [39]. After 1 hour of recovery, pups were exposed to hypoxia (8% oxygen balanced with nitrogen) for 90 minutes in purpose-built multi-chambers [14] which was made up with six multi-chambers, one gas reservoir bag, and one gas monitor with a built in gas pump. The total volume of the system is approximately 3.5 L. The system was flushed with more than four times of system volume of the mixed gases before the closed gas delivery circle system was established. Soda lime and silica gel (Merck, Leicestershire, UK) was used in the chambers to eliminate CO_2_ and H_2_O generated by animals. The concentration of oxygen was measured continuously by an in-line gas analyser (Air Products Model No. 439Xe, Surry, UK). Immediately after the hypoxic insult, the pups were randomly allocated to be exposed to either 70% argon or 70% N_2_ balanced with oxygen for 2 hours in the purpose-built, close-circuit delivery system mentioned above. The pups were sacrificed and their brains harvested day 1 or day 28 after gas treatment.

### Administration of wortmannin and U0126

Rats were given PI3K inhibitor wortmannin (16 μg/kg) [40], and Erk1/2 inhibitor U0126 ((1,4-diamino-2,3-dicyano-1,4-bis (2-aminophenylthio) butadiene; 1 nmol for 5μL; Cellsignalling, UK) through intra-cerebral injection [41] 30 min before hypoxic-ischaemic treatment.

### TUNEL staining

24 hours after HI, the presence of dead cells in the frontal cortex was detected an ApopTag® Fluorescein In Situ Apoptosis Detection Kit (S7110, Millipore, Bedford, MA) according to manufacturer's instructions.

### *In vitro* Nrf2 siRNA transfection and PI3K, ERK1/2 and m-TOR inhibition

SiRNA targeting rat Nrf2 (sc-37030, SantaCruz, USA) was administered to cultured neurons in a dose of 20nM, scrambled siRNA served as negative control. Cells were incubated with siRNA for 6 hours at 37°C in humidified air containing 5 % CO_2_, after which they were removed and replaced with experimental medium followed by OGD treatment. Other cohort cultures were treated with m-TOR inhibitor rapamycin (50 nmol/l) or vehicle (Tocris, Abingdon, UK) for 30 minutes before OGD treatment.

### *in vitro* cell viability

The viability of cells was assessed using a colorimetric MTT assay (Merck KGaA, Darmstadt, Germany).

### Determination of reactive oxygen species (ROS) *in vitro* by flow cytometry

ROS production was monitored by the measurement of superoxide (O2·–) and hydrogen peroxide (H_2_O_2_) generation using the fluorescent dyes dihydroethidium (DHE) [42] and carboxy-dichlorodihydrofluorescein diacetate (carboxy-DCF-DA)[43]. Cells were incubated in DHE (2μM) and carboxy-DCFDA (2μM) for 30 minutes at 37°C in the dark. The cells were washed with phosphate-buffered saline (PBS). Immunofluorescence intensity was acquired and analysed using flow cytometry (FACSCalibur; Becton Dickinson, Sunnyvale, CA). Each assay included at least 10,000 gated events.

### Immunohistochemistry

For *in vitro* fluorescence staining, cells were fixed in 4% paraformaldehyde. Cells were then incubated in 10% normal donkey serum in PBS-Tween 20 and then incubated overnight with either rabbit anti-p-m-TOR (1:200, Cell Signaling, Massachusetts, USA), rabbit anti-Nrf2 (1:200, Abcam), rabbit anti- NQO1 (1:200, Abcam), rabbit anti- cleaved caspase-3 (1:200, Cell Signalling, Massachusetts, USA), or mouse anti-α-tubulin (1:200, Sigma-Aldrich), followed by incubation with secondary antibody for 1 hour. For dual fluorescence labelling, cell samples or brain sections were incubated with the first primary antibody overnight, followed by its ascribed secondary antibody and then the second primary antibody incubation with subsequent secondary antibody. For *in vivo* fluorescence staining, the pups were sacrificed and transcardially perfused with 4% paraformaldehyde in heparinised PBS. The brains were then removed and fixed with 4% paraformaldehyde in PBS. They were then dehydrated in a 30% sucrose solution overnight before cryosectioning into 25 μm slices. Coronal sections were harvested between approximately -2.5 mm and -3.7 mm from bregma (relative to the adult rat brain). The brain sections were blocked with 3% normal donkey serum (NDS) (Millipore, Massachusetts, USA) in PBS with 0.1% Triton (PBS-T) added to the sections for 1 hour to block non-specific binding sites and to permeabilise the cell membrane. After blocking and permeabilisation, the sections were incubated overnight at room temperature with either rabbit anti-Nrf2 (1:200, Abcam, Cambridge, UK), rabbit anti-NQO1 (1:200, Abcam) or rabbit anti- SOD1 (1:200, Abcam) followed by rhodamine- or fluorescein isothiocyanate (FITC) conjugated secondary antibodies (Millipore, UK). The slides were counter-stained with nuclear dye DAPI and mounted with Vectashield mounting medium (Vector Laboaratories). Ten fields at ×20 view were first photographed using an AxioCam digital camera (Zeiss, Welwyn Garden City, UK) mounted on an Olympus BX60 microscope (Olympus, Middlesex, UK) with Zeiss KS-300 software. Staining was quantified using ImageJ software (U.S. National Institutes of Health, Bethesda, MD, USA). Fluorescent intensity was calculated as percentage of the mean value for naïve controls.

### Assessment of morphology of neurons through cresyl violet staining

Rats were anesthetised with sodium pentobarbital (100 mg/kg, intra-peritoneal) and perfused trans-cardially with paraformaldehyde (4%) in phosphate buffer (0.1 M). The brains were removed and placed in 4% paraformaldehyde in 0.1 mol/L phosphate buffer overnight. The brains were dehydrated and embedded in wax. Coronal sections of 5 mm were harvested at approximately -3.6 mm from the bregma relative to adult brain and then stained with 0.5% cresyl violet. The microphotograph was taken at 20× using a BX-60 light microscope (Olympus, Southall, UK) attached with a digital camera (Zeiss, Gottingen, Germany). The following morphologic criteria were used to analyse apoptotic or necrotic cell death in the cerebral cortex [44]. Necrotic or apoptotic cells were identified by dark-stained, shrunken nuclei that were spherically shaped with loss of nuclear membrane integrity. The total number of healthy cells that appeared in the cortex in cresyl violet staining slices were counted in a blinded manner and their mean value used for data analysis.

### Assessment of brain infarction through cresyl violet staining

The coronal sections (5 mm) from rats that received 90 mins hypoxic insult were selected from each pup to match predefined brain regions relative to the bregma (+2 mm, +1 mm, 0 mm, -1 mm, -2 mm and 5 mm) relative to adult brain. Once identified, each slice was photographed and the size (arbitrary unit) of the healthy matter of both hemispheres was calculated with data analysis software (ImageJ version 1.31; National Institutes of Health image software, Bethesda, MD) in a blinded manner. The infarction size was calculated with a formula of [(left hemisphere-right hemisphere)/left hemisphere] (%). These data were used to plot curves and the area under curve calculated to indicate the infarction volume (arbitrary unit).

### Enzyme-linked immunosorbant assay (ELISA)

Rat brain TNF-α and IL-6 concentration was measured by ELISA (Rat TNF-α and IL-6 ELISA kits, Invitrogen, UK).

### Assessment of malondialdehyde (MDA) level

The amount of lipid peroxide was measured as the production of MDA using MDA assay kit (Sigma-Aldrich, UK).

### Determination of total glutathione (GSH and GSSG)

Total glutathione (GSH and GSSG) was measured in brain homogenates using glutathione assay kit (Sigma-Aldrich, UK).

### Statistical analysis

Data is presented as mean ± standard deviation (S.D). Comparison between treatment groups was analysed by one-way ANOVA followed by post hoc Student Newman–Keuls test (GraphPad Prism 5.0 Software). Data with *p* values of <0.05 were considered statistically significant.
